# Three-week hypofractionated radiotherapy in early glottic cancer—a single institution retrospective study

**DOI:** 10.3332/ecancer.2022.1381

**Published:** 2022-05-04

**Authors:** Arun Sankar Sudha, Ravikumar Rejnish Kumar, Milan Anjanappa, Cessal Thomas Kainickal, Aleyamma Mathew, Ramadas Kunnambath

**Affiliations:** 1 Department of Radiation Oncology, Regional Cancer Centre, Thiruvananthapuram 695011, Kerala, India; 2 Department of Epidemiology & Biostatistics, Regional Cancer Centre, Thiruvananthapuram 695011, Kerala, India

**Keywords:** hypofractionation, glottic cancers, radiotherapy

## Abstract

**Background:**

Radiotherapy is a standard treatment option for early glottic carcinoma (stage I and II) with a fraction size of 2–2.2 Gy over 5–7 weeks. This study evaluates the outcome and prognostic factors of a 3-week hypofractionated treatment in early glottic malignancy.

**Materials and Methods:**

The case records of 329 eligible patients with stage I and II glottic carcinoma recorded at the institution from 2003 to 2008 were retrospectively analysed. All patients were treated in a Cobalt-60 machine to a dose of 52.5 Gy in 15 fractions (3.5 Gy/fraction) over 3 weeks.

**Results:**

Eighty-three percent had stage I disease. The local control rate at 5 years was 91.9%. On univariate analysis, stage I and II patients without subglottic extension had better local control. Disease extension to the subglottis fared poorly on multivariate analysis. After salvage treatment, the 5-year disease-free survival rate was 96.1% and the functional larynx preservation rate was 94.9% for stage I and 83.9% for stage II. The rate of severe complications was 2.1%.

**Conclusion:**

Comparable results with low morbidity are achievable with a 3-week hypofractionation in early glottic cancers and it offers better patient convenience.

**Highlights:**

- In early glottic cancer, hypofractionated radiation provides excellent local control.
- Subglottic extension is a poor prognostic factor.
- 5-year disease-free survival rate of 96.1%.
- 5-year functional larynx preservation rate of 94.9%.
- Severe complication rate of 2.1%.

## Introduction

Early glottic squamous cell carcinoma can be treated effectively by either radiotherapy or laser surgery with comparable outcomes [1]. The treatment choice often depends on the expertise available and patient preference. Although the results are very much similar, the voice quality is better with radiation [2, 3]. However, laser surgery has the advantage of being a single-day procedure, while radiation treatment is protracted over several weeks.

Various fractionation schedules are practiced all over the world with near equivalent results. Conventionally, 1.8–2 Gy/fraction is delivered to a total dose of 60–70 Gy over a protracted course of 6–7 weeks. Alternatively, fraction sizes of more than 2 Gy have also been used to deliver a curative intent dose with overall treatment time (OTT) reduction and modest improvement in local control rates. The use of 2.25 Gy fractions showed superior local control without adverse reactions in a randomised study from Japan [4]. However, very few centres follow a dose schedule with more than 3 Gy/fraction to reduce the OTT further [5–7]. Such schedules would be particularly useful in high-volume centres with long waiting lists to start radiation and also more convenient to the patient. The concern expressed over using high dose per fraction is that it may lead to increased late complications and limit its general acceptability [8].

The purpose of this study is to analyse the treatment outcome of a 3-week hypofractionation schedule of radiation at 3.5Gy/fraction (52.5 Gy in 15 fractions) in the treatment of early glottic cancers, late complications and to determine the prognostic factors.

## Materials and methods

The case records of patients with early glottic cancer (AJCC 7^th^ ed, cT1 and cT2 N0 M0) recorded between 2003 and 2008 were taken up for this retrospective analysis after the Institutional Review Board’s approval. Four hundred and twenty case records were available, of which on restaging, 73 were found to be advanced laryngeal cancers and excluded. Sixteen patients were treated with radiotherapy dose schedules other than 52.5 Gy in 15 fractions, and one patient with histology of spindle cell sarcoma and one patient with no follow-up details were excluded. Thus, 329 patient case records were taken for analysis. Pretreatment evaluation consisted of physical examination, endoscopy and biopsy, chest X-ray, blood haematology and biochemistry. A CT scan of the neck was performed based on physical examination findings and physician preference.

The median age of the population was 60 years. There were 319 males and 10 females. The majority of them had stage I (87%) disease. Patient characteristics are detailed in Table 1. Radiotherapy was planned in the supine position, using thermoplastic immobilisation. Lateral parallel-opposed pair fields were used, except in patients with short neck, for whom anterior oblique fields were used. The radiotherapy portal extended from an inferior border of the hyoid to the inferior border of the cricoid cartilage. The posterior limit of the portal was set at the anterior one-third of the vertebral body. A 3-mm bolus was applied to the region of the anterior commissure (AC) in patients who had lesions close to or involving the AC. Contours were taken at the level of the isocentre, which were digitised, and two-dimensional planning was carried out using the Plato planning system. Wedges were used as compensators in most patients and the dose was prescribed at the ICRU point. All patients were treated in a Cobalt unit, 5 days a week, for 3 weeks, to a total dose of 52.5 Gy in 15 fractions (3.5 Gy/fraction). The treatment characteristics are detailed in Table 2.

### Biological effective dose (BED) calculation

We calculated the BED for our treatment schedule using the following equation:

BED = Total dose (1+dose per fraction/(*α*/β))

With an *α*/*β* ratio of 10 for tumour control, the BED is 70.87 Gy_10_.

And for late complications, with an *α*/*β* ratio of 3, the BED is 113.75 Gy_3_.

The mean radiation duration was 19.7 days (range: 17–32 days). Three patients had a radiation gap of more than 3 days. The median field size used was 6.2 × 6.3 cm and the median field area was 39 cm^2^ (range: 30–68 cm^2^). The treatment characteristics are outlined in Table 2.

Patients were followed up with physical examination and laryngoscopy every 3–4 months for the first 2 years, 6 monthly till 5 years and yearly thereafter. Imaging studies were carried out in cases of clinical suspicion of recurrence or for evaluation of persistent oedema. Biopsy was done only when the clinical suspicion of recurrence was high.

### Statistical analysis

The end points analysed were local control, disease-free survival (DFS), overall survival (OS), cause-specific OS and laryngeal function preservation at 5 years. Local recurrence was defined as failure in the larynx, node and both. DFS events were described as any recurrence or death due to any cause. Any death was considered an event for estimating OS and death due to disease for cause-specific OS. Survival with laryngeal function preservation included death from laryngeal cancer, salvage laryngectomy and tracheostomy as events. All outcomes were measured from the date of registration at the hospital. Univariate analysis of various patient and treatment related factors on the outcome were carried out using log-rank test. Multivariate analysis was carried out using the Cox regression method. Kaplan–Meier estimates were used to calculate the survival curves.

## Results

### Local control

The median follow-up of surviving patients was 86 months (5–149 months) and 77.4% of the total number had more than 5 years of follow-up. At 5 years, the local control rate was 91.9%. There were a total of 25 recurrences. Among them, 19 were local (stage I: 13; stage II: 6), 5 nodal (stage I: 4; stage II: 1) and 1 locoregional (stage II) relapse. Median time to recurrence was 13 months (0–57). Three-quarters of the recurrences occurred within 24 months.

**Figure 1.**
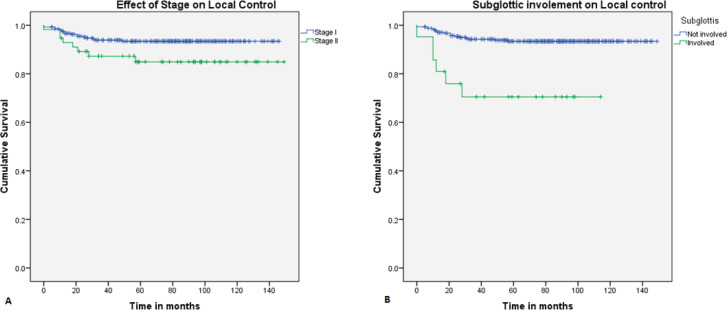
Kaplan–Meier curve showing difference in (a): local control with stage and (b): subglottic involvement.

On univariate analysis, the local control was better in stage I (94.2%) compared to stage II (84.8%) (Figure 1a), which is statistically significant (*p* = 0.02). Patients with subglottic involvement had poor local control (70.5%) (Figure 1b) compared to patients without subglottic involvement (93.7%) (*p* = 0.0001). Furthermore, patients treated with a field size of ≤39 cm^2^ had a favourable local control (94.6%). Other factors, like age, supraglottic involvement, impaired vocal cord mobility, anterior commissure involvement, tumour grade, and duration of treatment, did not show any statistical difference (Table 3). In multivariate analysis, only subglottic involvement retained significance (*p* = 0.024)

### Treatment of recurrence and disease-free survival

Of the 25 recurrences, 14 patients underwent salvage surgery (13 total laryngectomy and 1 neck dissection). Two patients further developed recurrence (one stomal and one nodal recurrence each). Among the patients who did not undergo salvage surgery, one patient remained disease-free after chemotherapy for nodal recurrence. At 5 years, the DFS was 96.1%. None of the factors influenced DFS (Table 4).

### Overall survival

There were a total of 46 deaths, and at 5 years, the OS was 86.5%. Twelve patients died due to the disease, 13 patients due to a second malignancy, 10 of other causes, and in 11, the cause was unknown. The disease-specific OS was 96.4%.

### Laryngeal function

A total of 25 patients had tracheostomy (Table 4). Two patients had an elective tracheostomy prior to radiotherapy, which was subsequently closed upon treatment completion. The 5-year functional larynx preservation rate was 93%, but it was 94.9% for stage I and 83.9% for stage II. The 5-year survival with functional larynx was 90.8%, but it was 92.8% for stage I and 81.2% for stage II.

### Complications

Laryngeal oedema or hoarseness of voice and dyspnoea were noted in 42 patients (12 were due to the disease). The symptoms were resolved with anti-inflammatory/steroids in 24 patients. A total of six patients had grade 3 and 4 toxicity (three had chondronecrosis and three had tracheostomy), with a 5-year actuarial rate of 2.1%. It was observed that patients who developed chondronecrosis were active smokers after treatment. The median field size among patients with and without complications was not different (39 cc in both the groups). Cerebrovascular accident was noted in one patient who died of it 5 years after the treatment for glottic carcinoma.

### Second malignancy

Eighteen patients developed second malignancies. Six patients developed head and neck cancers, five developed lung malignancy and two had oesophageal second primaries. The remaining five had cancers in other sites.

## Discussion

Our series further underscores that radiotherapy offers effective local control in early glottis carcinoma. In addition, this 3-week treatment schedule can achieve comparable results with that of 4–7 week treatment schedules practiced across the globe. Various factors have been described which affect the local control. Among them, fraction size, total dose, OTT, T-stage, anterior commissure involvement and subglottis extension appear significant.

There is considerable heterogeneity in the available literature regarding the dose per fraction and total dose delivered. Some of the various dose fractionation schemes and local control rates reported in literature are outlined in Table 5. Fraction size has been regarded as one among the important prognostic factors [9, 23–25]. Fraction size of <2 Gy has an inferior local control rate [10, 13, 25, 26]. In a randomised study by Yamazaki *et al* [4], the 5-year local control for 2 Gy per fraction was 77% and for 2.25 Gy/fraction was 92%. Most of the results with a 2 Gy or less dose per fraction had an overall prolongation of time as well. This could also be a contributing factor for poor disease control. In the randomised non-inferiority trial by the Japanese Clinical Oncology study group (JCOG 0701), accelerated fractionation (2.4 Gy/fraction) was compared to standard fractionation. The cumulative incidences of local failure at 3 years for SF/AF were 15.9%/10.3%. No significant difference was observed in the 3-year OS between SF and AF. Grade 3 or 4 acute and late toxicities developed in 22 (12.4%)/21 (11.5%) and 2 (1.1%)/1 (0.5%) in the SF/AF arms, respectively [21]. In another randomised study, KROG-0201, the HYPO arm (63–67.5 Gy at 2.25 Gy/fraction) was found to be at least non-inferior to the CONV arm (66–70 Gy at 2 Gy/fraction). The 5-year local progression-free survival was 88.5% and 77.8% (HR = 1.55, *p* = 0.213) respectively, with no difference in the toxicity profile [27]. A meta-analysis of 1,762 patients with early stage glottic carcinoma showed that altered fractionation radiation was associated with 38% and 60% fewer local failure events in a pooled analysis of randomised and retrospective studies respectively. Hypofractionation was superior to conventional radiation (HR= 0.55, *p* = 0.02) [28].

Reports of utilisation of fraction size more than 3 Gy in clinical practice are very few. Gowda *et al* [5] reported their experience with a 3-week treatment schedule of 3.12 and 3.28 Gy per fraction, delivering a total dose of 50 and 52.5 Gy, respectively, in 16 fractions. The local control rate was 93% and the ultimate local control was 96% at 5 years with very minimal late serious complications. Voet *et al* [14], in their series, compared six different fractionation schedules (60 Gy (3.25 Gy × 20), 62 Gy (3.1 Gy × 20), 61.6 Gy (2.8 Gy × 22), 60 Gy (2.4 Gy × 25), 66 Gy (2 Gy × 33) and 60 Gy (2 Gy × 30)) The local control rate at 5 years was 93%, 90%–91% and 83%–85% for fraction sizes >3, 2–3 and 2 Gy, respectively. The reported grade III–IV complication rate at 5 years for >3 Gy/fraction was 5.3% and for 2–3 Gy/fraction was 1.8%–3.1%. The authors also point out in that the complication rates were high patients who continued to smoke. In a similar retrospective cohort comparison study by Tata Memorial Hospital, the group with >3 Gy/fraction (50 Gy (3.3 Gy × 15) and 55 Gy (3.43 Gy × 16)) had a comparable local control with that of <3 Gy/fraction (60 Gy (2.5 × 24) and 62.5 Gy (2.5 × 25)) at 10 years (88.4% versus 84%) [18]. The reported rate of complications was not significant between the two groups.

The reluctance to accept a high dose per fraction treatment is the fear of increased late complications. The severe late complications described in the literature with radiation range from 0 to 5.4% for various fractionation regimens (Table 5). The reported rate of complications for >3 Gy/fraction is relatively low as described earlier. Dinshaw *et al* [6], in their study comparing different regimens, did not observe any increase in late complications for high dose per fraction (3.33 Gy/fraction). The serious late complications in our experience are low (2.1%) and comparable with most other studies. A corresponding reduction in total dose delivered can adjust for the late effects of high dose per fraction.

Prolonged treatment time has shown to negatively impact treatment outcomes in head and neck squamous cell carcinoma [29]. The same has been demonstrated in T1 glottic cancers as well [9, 26, 30, 31]. In order to overcome the accelerated repopulation, occurring during radiation, an estimated dose of 0.1–0.48 Gy/day is required to compensate for treatment extending beyond 28 days [32–34]. Each 1-day extension in overall treatment results in 1.3% loss in local control [34]. The advantage of a 3-week regimen helps to overcome the problem of accelerated repopulation. Finally, dose per fraction, total dose and OTT have a complex interplay and each variable cannot be considered exclusively in explaining their role in tumour control.

Anterior commissure involvement has been demonstrated as a high-risk factor for local recurrence [11, 35, 36]. Hirota *et al* [37] reported a 5-year local control of 57.6% and 89.9% with and without AC involvement, respectively. The local control was superior if the dose delivered was 70–72 Gy rather than 60–62 Gy. In a large series from Italy with 1087 patients, the local control was 78% (AC involved) and 87% (AC-free) at 5 years [17]. In another multicentre study, anterior commissure involvement negatively impacted the locoregional control with an HR of 1.51 [38]. The possible reasons for poor outcome may be due to unnoticed thyroid cartilage involvement or subglottic extension of the disease. Second, the neck is relatively thin near the region of AC and this lack of tissue will reduce the dose build up [39]. On the contrary, some of the studies have not found significant differences in the local control when AC is involved [5, 10, 40–43]. In our series, AC involvement was not a significant prognostic factor. This can be explained by the department treatment policy to use a 3–5 mm bolus over the region of AC for patients with an anterior tumour, close to or involving AC and hence improving the dose coverage.

Furthermore, at our centre, we prefer to use Cobalt-60 external beam radiation for treating early glottic cancers. For a small field size, compared to Co-60 beam, the dose distribution near the surface is poor for 6 MV photon [44]. Also, there is loss of charged particle equilibrium at the air–tissue interface which is more pronounced for high-energy beams, leading to a lower dose distribution [45]. Spirydovich *et al* [46], in their mathematical model, demonstrated that at least 5% volume of a 3.5 cc hypothetical tumour received less than 86% of the maximum tumour dose. However, the reported clinical experience does not show any drop in local control rates. The 5-year local control rates for T1 glottic cancers treated with 6 MV photon ranged from 89% to 93% [5, 30, 40, 47–49].

Several authors have reported that the subglottic extension of tumour is a prognostic factor [6, 11, 16, 42]. Warde *et al* [42] reported a 1.7 times higher chance of local recurrence in patients with disease extension to subglottis. In another series with 280 patients, the 5-year local control rate was 63% and 81% if subglottic extension was present and absent, respectively [16]. In our study as well, patients with subglottis extension had inferior local control. Impaired vocal cord mobility has also been reported to have poorer local control rates [20, 50]. Dixon *et al* [20], in a study of accelerated hypofractionation in T2 glottic cancer, reported worse 5-year local control for patients with impaired vocal cord mobility (T2b: 70.8%) as compared to supra/subglottic extension (T2a: 88.8%). In our study, patients with impaired vocal cord mobility showed a trend towards worse local control. The limited number of patients in our study with T2 stage may limit the validity of the analysis.

A few authors have noted worsening of local control with poorly differentiated carcinoma. In a study of 478 patients, which also included supraglottic tumours, a significantly higher incidence of events was noted with lower tumour differentiated tumours [51, 52]. However, as in our study, many studies have shown no difference in local control attributed to tumour differentiation [36, 48, 53].

The importance of field size in local control was demonstrated by Harwood *et al* [54]. The local recurrence rate improved from 18% to 9% when the field size was increased from 5 × 5 cm to 6 × 6 cm with a free set up and Co-60 therapy unit. However, Teshima *et al* [55] in a prospective randomised study did not find any difference in local control with a 5 × 5 cm and 6 × 6 cm field using a 4 MV photon. This, as explained by the authors, could be due to improved immobilisation. Also, Co-60 produces a larger penumbra and this necessitates a larger field size. Fein *et al* [30] reported a 2-year control rate of 90% for irradiated area <36 cc and 86% for ≥36 cc. Similar to this, few other studies did not find field size impacting local control [6, 17, 18, 40]. In the present analysis, field area >39 cc had a poor control. This could be due to the T2 disease in which comparatively larger fields were used.

Field size has an influence on the radiation toxicity, particularly laryngeal oedema. Teshima *et al* [55] reported an increase in persistent arytenoid oedema (not requiring medication or surgery) lasting more than 6 months in the group treated with 6 × 6 cm. Cellai *et al* [17] reported 19% complications (minor and major) for field size area >49 cc and major complications (requiring tracheostomy or chondritis) was 3.5%. For fields less than 49 cc, the overall complications were 5% and major complications were less than 1%. Several other investigators have also reported an increase in laryngeal oedema when field area was ≥36 cc [6, 56]. Le *et al* [11] showed a trend in increased complications 1% versus 2.6% for field area ≤30 cc versus larger field size.

Radiation to the neck has been shown to increase the carotid artery stenosis and carotid sparing intensity-modulated radiotherapy (CS-IMRT) has been shown to reduce the dose to radiation to them. However, long-term prospective data would be required to show the benefit of CS-IMRT [57]. This 3-week hypofractionated schedule, delivered using CS-IMRT, is a potentially attractive option worth exploring in the future.

Treatment failure after radiation is quite low and manageable with surgical salvage. In our series, nearly half of the recurrences were salvaged surgically. Finally, larynx preservation rate is an important factor to judge a treatment outcome. The larynx preservation rate in this study is 93%, which is comparable with the reported rates of 87%–97% [14, 17, 18, 47].

The drawbacks of this study are that being a retrospective study, the adverse effects may be under reported. Less serious potential adverse effects, like soft tissue fibrosis or dysphagia, were not consistently documented. Moreover, quality of voice was not formally tested or documented. 77% of patients completed 5 years of follow-up, which although respectable, is far from ideal, and may impact the results of this study. The strength of the study is that it describes the results of one of the largest single-centre series of patients treated with altered fractionated radiotherapy. Its adoption would be more convenient to the patient in terms of reduction in hospital visits and medical costs, and also in helping streamline access to treatment in high-volume centres, especially in developing countries.

## Conclusion

The 3-week radiation schedule for early glottis carcinoma is comparable to other more protracted schedules and is associated with minimal complication rates. This short hypofractionation schedule could be more convenient to the patient and has potential as a treatment option for early glottis carcinoma.

## Conflicts of interest

None of the authors have any conflict of interest in the publication of this manuscript.

## Funding statement

This research received no specific grant from any funding agency in the public, commercial or not-for-profit sectors.

## Figures and Tables

**Table 1.** Patient characteristics.

	*N* = 329
Age (median)	60 (17-89) years
**Sex**	
Male Female	319 (97%) 10 (3%)
**Habits**	
Smokers Tobacco chewers Alcohol consumption	282 (85.7%) 50 (15.2%) 153 (46.5%)
Co-morbidities	100 (30.3%)
Anterior Commissure involvement	158 (48%)
Supraglottis involvement	14 (4.3%)
Subglottis involvement	21 (6.4%)
Impaired vocal cord mobility	21 (6.4%)
**T stage**	
1a 1b 2	225 (68.4%) 48 (14.6%) 56 (17%)
**Composite stage**	
Stage I	273 (83%)
Stage II	56(17%)
CT scan	171 (52%)
Histology Squamous cell carcinoma	329 (100%)
**Grade**	
Grade 1 Grade 2 & 3	191 (58%) 138 (42%)

**Table 2.** Treatment characteristics.

	*N* = 329
**RT planning**	
Parallel pair Oblique pair	325 (98.8%) 4 (1.2%)
**Bolus over AC**	
None Present	69 (21%) 260 (79%)
**Field size**	
30–36 sq.cm >36 sq.cm	100 (30.3%) 229 (69.7%)
**RT duration**	
Up to 21 days >21 days	286 (87%) 43 (13%)

(AC-Anterior Commissure, RT-Radiotherapy)

**Table 3.** Univariate analysis of prognostic factors on local control and DFS.

		Local Control			DFS		
Factors		*N* = 25	Local control (%)	*p* value	*N* = 12	DFS (%)	*p* value
Stage	Stage I	17	93.9	0.042	9	96.5	0.469
	Stage II	8	84.9		3	94	
Age	≤60	14	91.4	0.66	7	95.7	0.662
	>60	11	92.4		5	96.5	
Supraglottic involvement	Present	0	100	0.269	0	100	0.45
	Absent	25	91.5		12	95.9	
Subglottic involvement	Present	6	70.5	0.001	2	90.5	0.123
	Absent	19	93.4		10	96.5	
Impaired VC mobility	Present	2	92.1	0.06	1	93.3	0.817
	Absent	23	93.4		11	96.3	
Anterior commissure	Present	14	90.9	0.404	6	96.10	0.893
	Absent	11	92.8		6	96.10	
Tumor grade	Grade 1	15	91.5	0.816	7	96.10	0.969
	Grade 2&3	10	92.5		5	96.20	
Treatment duration	≤21 days	20	92.7	0.29	1	95.90	0.623
	>21 days	5	86.6		11	97	
Field size	≤39cc	10	94.6	0.032	4	97.9	0.056
	>39cc	15	87.9		8	93.4	

DFS, Disease free survival

**Table 4.** Various cause for tracheostomy.

Cause	Number (*n* = 25)
Before treatment(airway narrowing)	2
Recurrent/Persistent disease	19
Radiation toxicity Chondronecrosis Subglottic stricture Persistent breathing difficulty	1 1 2

**Table 5.** Various studies with reported fractionation, treatment time, local control and severe complication.

Study	*N*	Fraction size (Gy)	Total dose (Gy)	OTT (days)	Follow up	Local control	Severe complication
Mendenhall [9]	304 (T1–T2)	2.1–2.25	56–67	NA	5	T1-93% T2b-72%	1.6%
Burke [10]	102 (T1–T2)	1.67–3.33	50–74.4 Median 65	49	5	80%–92%	2%
Le [11]	398 (T1–T2)	1.3–2.4	46.6–76	50	5	82%	1.8%
Reddy [12]	114 (T1)	1.8–2	60–70	42–60	5	82%	1.7%
Yu [13]	126 (T1)	2.5 2.25 2	50 65.25 66	26–46	10	76% 84%(>2Gy)	Nil
Voet [14]	383 (T1)	2–3.25	60–65	22–>40	5	89%	1.8%–5.3%
Dinshaw [6]	676 (T1–T2)	3.33 2.5 2–2.5	50 60–62.5 55–60	22	10	T1-82% T2-57%	<1%
Lee [15]	128 (T1–T2)	2 1.2–1.6 (b.i.d)	66 Gy 60–74.2	NA	3	T1-86% T2-68%	2%
Gowda [5]	200 (T1)	3.28 2.12	52.5 50	21–26	5	93%	<1%
Garden [16]	230 (T2)	2.06–2.26 2 1.2(b.i.d)	66–70 32–75 74–80	45	5	72%	4%
Cellai [17]	831 (T1)	≤2 >2.4	<61 >65	<45–>60	10	83%	0.7%
Yamazaki [4]	180 (T1)	2 2.25	60–66 56.25–63	NA	5	77% (2Gy) 92% (2.25Gy)	Nil
Laskar [18]	652(T1)	3.33 3.43 2.5	50 55 60	NA	10	84% (<3Gy) 86.1% (>3Gy)	1%
Ermis [19]	132 (T1–T2)	2.75	55	28	5	85.6%	2.2%
Dixon [20]	112 (T2)	3.28	52.5	22	5	82%	1.8%
JCOG0701 [21]	370 (T1–T2)	2.4 2	60–64.8 66–70	NA	3	89.7% 84.1%	0.5% 1.1%
Salas [22]	138	2.25 2	63 70	40 51	10	83.9% 83.7%	1.5% 1.4%
Present study	329 (T1–T2)	3.5	52.5	19	5	91.9%	2.1%

OTT, Overall treatment time
